# Novel Variants in the *CLCN1*, *RYR2*, and *DCTN1* Found in Elderly Japanese Dementia Patients: A Case Series

**DOI:** 10.3390/geriatrics6010014

**Published:** 2021-02-07

**Authors:** Atsushi Hori, Tomohiko Ai, Miwa Isshiki, Yumiko Motoi, Kouji Yano, Yoko Tabe, Nobutaka Hattori, Takashi Miida

**Affiliations:** 1 Center for Genomic and Regenerative Medicine, Juntendo University Graduate School of Medicine, Tokyo 113-8424, Japan; a.hori.vb@juntendo.ac.jp (A.H.); ko-yano@juntendo.ac.jp (K.Y.); 2 Department of Clinical Laboratory Medicine, Juntendo University Graduate School of Medicine, Tokyo 113-8424, Japan; m-issiki@juntendo.ac.jp (M.I.); tabe@juntendo.ac.jp (Y.T.); tmiida@juntendo.ac.jp (T.M.); 3 Krannert Institute of Cardiology, Indiana University School of Medicine, Indianapolis, IN 46202, USA; 4 Department of Neurology, Juntendo University Graduate School of Medicine, Tokyo 113-8424, Japan; motoi@juntendo.ac.jp (Y.M.); nhattori@juntendo.ac.jp (N.H.)

**Keywords:** dementia, Alzheimer’s disease, genetic test

## Abstract

Dementia has an enormous impact on medical and financial resources in aging societies like Japan. Diagnosis of dementia can be made by physical and mental examinations, imaging tests, and findings of high abnormal proteins in cerebrospinal fluids. In addition, genetic tests can be performed in neurodegenerative diseases such as Alzheimer’s disease (AD), frontotemporal dementia (FTD), and Parkinson’s disease (PD). In this case series, we presented three cases of dementia with unknown causes who carry novel variants in the genes associated with neurodegenerative diseases. Three patients (Patients 1, 2, and 6) were found by screening 18 dementia patients using a gene panel including 63 genes. The age of onset for Patient 1 was 74 years old, and his father had PD and mother had AD. The age of onset for Patient 2 was 75 years old, and her mother had AD. The age of onset for Patient 6 was 83 years old, and her father, two sisters, and daughter had dementia. The Mini-Mental State Examination produced results of 20, 15, and 22, respectively. The suspected diagnosis by neurological examinations and imaging studies for Patients 1 and 2 was AD, and for Patient 6 was FTD. Patient 1 was treated with donepezil; Patient 2 was treated with donepezil and memantine; and Patient 6 was treated with donepezil, galantamine, and rivastigmine. The three rare variants identified were: *CLCN1*, encoding a chloride channel, c.2848G>A:p.Glu950Lys (Patient 1); *RYR2*, encoding a calcium releasing ryanodine receptor, c.13175A>G:p.Lys4392Arg (Patient 2); and *DCTN1*, encoding a subunit of dynactin, c. 3209G>A:p.Arg1070Gln (Patient 6). The detected variants were interpreted according to the American College of Medical Genetics (ACMG) guidelines. The minor allele frequency for each variant was 0.025%, 0.023%, and 0.0004% in East Asians, respectively. The *DCTN1* variant found in Patient 6 might be associated with FTD. Although none of them were previously reported in dementia patients, all variants were classified as variants of unknown significance (VUS). Our report suggests that results of genetic tests in elderly patients with dementia need to be carefully interpreted. Further data accumulation of genotype–phenotype relationships and development of appropriate functional models are warranted.

## 1. Introduction

Dementia has an enormous impact on medical and financial resources in aging societies like Japan [1]. Although dementia might be a non-pathological manifestation of aging [2], dementia can be also associated with various types of neurodegenerative diseases such as Alzheimer’s disease (AD), Lewy body dementia, frontotemporal dementia, Parkinson’s disease, and hippocampal sclerosis [3,4]. Of these, it has been reported that AD is the leading cause of dementia.

Currently, AD is diagnosed based upon an A/T/N biomarker system consisting of abnormal amyloid and *Tau* proteins in cerebrospinal fluids (CSFs) as well as imaging studies such as positron emission tomography and MRI [5]. In addition, it has also been reported that some forms of AD are inheritable and caused by genetic abnormalities [6]. To date, more than 300 of variants in *PS1*/*PS2* and *APP* genes have been identified [7]. It has been theorized that accumulation of abnormal amyloid proteins produced by these variants are accountable for genetic AD [8]. Several genes such as *APOE4*, *TREM2*, *SORL1*, and *ABCA7* traits have also been reported to be risk factors of AD [9]. 

In addition, several clinical genetic tests for dementia are available: Invitae Combined Hereditary Dementia and Amyotrophic Lateral Sclerosis Panel containing 28 genes, and 3 genes for Alzheimer’s Disease (https://www.invitae.com/en/physician/tests/03502/ (accessed on 18 January 2021); https://www.invitae.com/en/physician/tests/03504/ (accessed on 18 January 2021)); Blueprint Genetics Dementia Panel containing 58 genes (https://blueprintgenetics.com/tests/panels/neurology/dementia-panel/ (accessed on 18 January 2021)); GeneDx Dementia containing 5 genes (https://www.genedx.com/test-catalog/disorders/dementia/ (accessed on 18 January 2021)). 

Although discovery rates regarding genetic testing in elderly dementia seems to be low [10], it could potentially be an adjunctive for diagnosis. We screened 18 Japanese patients with dementia of unknown cause using a gene panel of 63 genes associated with neurodegenerative diseases. We herein presented the clinical profiles of three patients in whom three novel variants were found and discussed the interpretation of those variants.

## 2. Material and Methods

### 2.1. Patient Recruitment

The study was conducted according to the criteria set by the Declaration of Helsinki and the study protocol was approved by an ethical committee in Juntendo University School of Medicine, Tokyo, Japan. The guardians of these participants provided consent on their behalf before participating in the study. Out of 100 dementia patients whose diagnoses were uncertain, we selected 18 patients whose genetic tests were negative for *PS1*/*PS2* and *APP* and who had positive family history of dementia. Dementia was diagnosed based upon medical examinations by neurologists using clinical dementia scores and imaging studies such as CT scans, MRIs, and SPECTs [5,11,12]. Due to the patient’s or the guardian’s wish, CSFs were not tested in most of the cases.

### 2.2. Genetic Analyses

Genomic DNA was extracted from lymphocytes using the QIAamp DNA Mini kit (QIAGEN, Germantown, MD) and stored at −80 °C until use. All coding exons of the following 63 genes that are potentially associated with dementia were analyzed: *MAPK1*, *TREM2*, *ABCA7*, *APP*, *PSEN1*, *PSEN2*, *APOE, HSPG2*, *FCN2*, *GRIN1*, *SORL1*, *MAPT*, *GRN*, *TDP-43*, *C9ORF72*, *VCP*, *FUS*, *SOD1*, *TARDBP*, *PFN1, SQSTM1*, *A2M*, *AAAS*, *ACE, CSF1R*, *DCTN1, DNMT1*, *EIF4G1*, *FBXO7*, *GBA*, *GCH1*, *GRN*, *HTRA2*, *LRRK2*, *MPO*, *PARK2*, *PARK7*, *PINK1*, *PLA2G6*, *POLG*, *PRKRA*, *PRNP*, *SLC6A3*, *SNCA*, *SNCB*, *TAF1*, *TYROBP*, *UCHL1*, *VPS35, KCNN2*, *KCNN3*, *ATP2A2*, *RYR2*, *CALM1*, *ATP2B1*, *SLC8A1*, *TRPC1*, *CLCN1*, *SCN4A*, *CACNA1C*, *KCNJ2*, *ATP13A2*, and *ATP1A3* [10]. 

Sequencing was performed with the Ion Torrent personal genome machine (PGM) sequencer as described previously (Thermo Fisher Scientific, Waltham, MA, USA) [13]. Briefly, primers for the genes were designed using the Ion Ampliseq Designer (Ion Torrent; Thermo Fisher Scientific, Waltham, MA, USA). Paired-end sequencing (2 × 400 bp) of the enriched library was performed using the Ion Torrent PGM sequencer. Resulting sequences were aligned to the human genome reference (hg19), and variants were identified by Torrent suite software v5.6.0., which was equivalent to GATK 4.0 (Broad Institute: https://gatk.broadinstitute.org/hc/en-us (accessed on 18 January 2021)). The generated Bam files were annotated with Ion Reporter Software v5.6.0. Detected variants were first inspected by Integrative Genomics Viewer (IGV, Broad Institute: http://software.broadinstitute.org/software/igv/ (accessed on 18 January 2021)), and were confirmed by Sanger sequencing using 3500 Genetic Analyzer (Thermo Fisher Scientific).

### 2.3. Variant Interpretation

Variants were interpreted according to the American College of Medical Genetics (ACMG) guidelines 2015 [14,15]. Minimal allelic frequency (MAF) in the general population was obtained from gnomAD v2.1.1. [16]. ClinVar [17] was used to screen benign and likely benign variants. Other resources used were: Online Mendelian Inheritance in Man (OMIM) [18]; Gene Reviews [19]; Monarch Disease Ontology (MonDO) [20]; ORPHANET [21]; and PubMed [22].

## 3. Results

### 3.1. Patient Background

Table 1 shows the list of patients. The average age during the genetic tests was 74.4 ± 9.8 years old (4 male and 14 females), and average age of onset (±S.D.) was 73.5 ± 8.7 years old. The average MMSE (Mini-Mental State Examination) score was 22.4 ± 4.1. All patients had family history of dementia. Nine patients (50.0%) had comorbid diseases including hypertension, diabetes mellitus (DM), and hyperlipidemia. Nine patients showed no AD features for MRIs and SPECTs (patients #1, 2, 3, 6, 15, 16, 19, 21, and 23). 

The sequencing using the gene panel detected rare genetic variants in patients #1, 2, and 6: *CLCN1* nucleotide change c.2848G>A, resulting in p.Glu950Lys; *RYR2* nucleotide change c.13175A>G, resulting in p.Lys4392Arg; and *DCTN1* nucleotide change c.3209G>A, resulting in p.Arg1070Gln, respectively.

### 3.2. Clinical Characteristics of the Patients with Rare Variants

The clinical characteristics of the three patients (#1, 2, and 6) whose genetic tests showed novel variants were described as below. 

**Figure 1:**
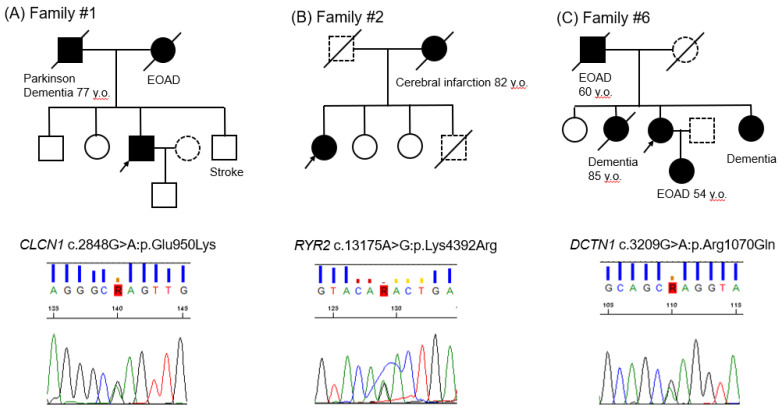
(**A**): Family #1; (**B**): Family #2; (**C**): Family #3. Pedigrees and Sanger sequencing results. Black arrows indicate the probands; closed symbols, dementia individuals; slash, deceased individuals; dotted symbols, unknown disease status. EOAD depicts early onset of Alzheimer’s disease.

Patient #1. The patient was a 78-year-old male. He has been suffering from Diabetes Mellitus (DM) for 10 years and is treating it with insulin. At the age of 73, he gradually developed memory loss. His CT scan showed mild diffuse brain atrophy and his MRI showed mild brain atrophy with lacunar infarction. His SPECT revealed hypoperfusion in surface areas in bilateral frontal, temporal, and lower parietal lobes, as well as in the cingulate gyrus and precuneus. One year later, he became incapable of money counting and shopping. His cognitive functions were impaired, and his alertness had begun to fluctuate. He started taking donepezil (3–5 mg). Three years later, his DaTscan showed pre-synaptic disturbance of dopaminergic neurons in substantia nigra. Although he did not show apparent tremors, he showed akinesia. His diagnosis seemed to mostly correspond to Lewy body dementia. The pedigree shows that his father was suffering from Parkinson’s disease and dementia at the age of 77, and his mother suffered from early-onset AD at the age of 67 (Figure 1A upper panel). The Sanger sequencing result shows *CLCN1* nucleotide change c.2848G>A, resulting in p.Glu950Lys (Figure 1A lower panel).

Patient #2. The patient was a seemingly healthy 77-year-old female. At the age of 73, she started having short-term memory loss. Her SPECT showed hypoperfusion areas in the bilateral frontal, temporal, and lower parietal lobes, as well as in the cingulate gyrus and precuneus, which might be consistent with AD. She has been treated with donepezil as a diagnosis of AD. She then developed hallucinations (e.g., wallpaper stains appeared to look like insects). She was treated with additional memantine. Six months later, she showed irritability, angriness, and suicidal thoughts. Her MMSE score decreased to 8 from 15 in three years. Her MRI showed only mild brain atrophy and lacunar infarction. Her DaTscan showed unremarkable findings. She has never had arrhythmia attacks. The pedigree shows that her mother suffered from cerebral infarction at the age of 82 (Figure 1B upper panel). The Sanger sequencing result shows *RYR2* nucleotide change c.13175A>G, resulting in p.Lys4392Arg (Figure 1B lower panel).

Patient #6. The patient was an 85-year-old female with a medical history of chronic kidney failure and hypertension. At the age of 76, she started having occasional urinary incontinence. Her MRI was normal except an incidental aneurysm of the left internal carotid artery. Her SPECT showed patchy hypoperfusion areas in the bilateral frontal lobes near Sylvian fissure, basal temporal lobes, and gyrus cinguli. Five years later, she developed short-term memory loss. Her MMSE score was 22. She has been treated with donepezil, galantamine, and rivastigmine. Three years later, she developed dyspnea, but her respiratory functions were preserved. She developed occasional illusions in the next two years. Her definite diagnosis remains uncertain. Her SPECT showed diffuse hypoperfusion in the frontal and bilateral lower temporal lobes. The pedigree shows that her father and daughter suffered from EOAD at the age of 60 and 54, respectively (Figure 1C upper panel). The Sanger sequencing result shows *DCTN1* nucleotide change c.3209G>A, resulting in p.Arg1070Gln (Figure 1C lower panel).

### 3.3. Classification of the Three Variants Based upon the ACMG Guidelines 

The variant *CLCN1* c.2848G>A:p.Glu950Lys was found in 0.025% of East Asian populations (MAF = 0.003% in a total of 282,374 alleles in gnomAD v2.1.1), and has not been reported in ClinVar. The variant *RYR2* c.13175A>G:p.Lys4392Arg was found in 0.023% of East Asian populations (MAF = 0.0016% in a total of 246,870 alleles in gnomAD). However, this variant has been reported in cases with catecholaminergic polymorphic ventricular tachycardia (CPVT) [14,23]. The variant *DCTN1* c.3209G>A:p.Arg1070Gln was not found in East Asian populations (MAF = 0.0004% in a total of 250,216 alleles in gnomAD), and has not been reported in ClinVar. In silico analyses using Sift, Polyphen, and Grantham [15] showed a pathogenic role of *CLCN1* c.2848G>A:p.Glu950Lys, but not the other two variants (Table 2). Thus, currently, these three variants are classified as VUS.

## 4. Discussion

In this case series, we presented three Japanese dementia patients who carried genetic variants associated with neurodegenerative diseases. We found two novel variants in the genes associated with neural diseases (*CLCN1* and *DCTN1*) and one *RYR2* variant that was previously found in cardiac arrhythmia syndrome. Although the interpretations of these variants were “variants of unknown significance (VUS)” according to the ACMG guidelines [15], our report might be useful for clinical practitioners in geriatric medicine.

Variants in the *CLCN1*, encoding a chloride channel, can cause Myotonia Congenita that usually causes muscle weakness and cramps in infants. Age of onset and symptoms vary depending upon inheritance trait—autosomal dominant (Thomsen disease) or recessive trait (Becker disease)—and possibly upon variants [24]. Patient #1 did not show any neuromuscular symptoms besides age-associated muscle atrophy. Variants in the *RYR2*, encoding a ryanodine receptor, can cause catecholaminergic polymorphic ventricular tachycardia (CPVT), a fatal arrhythmia. Although major subtypes of RYR in neuronal cells are RYR3 and RYR1 [25], RYR2 has been reported to be expressed in cerebellum Purkinje cells, the cerebral cortex, and the hippocampus [26]. Patient #2 has never had VT attacks. However, *RYR2* variants can be associated with abnormal Ca^2+^-handling in association with Alzheimer’s disease [27]. Variants in *DCTN1*, encoding a protein dynactin-1, can be associated with various neurodegenerative syndromes such as Perry syndrome, Amyotrophic lateral sclerosis, Charcot-Marie-Tooth disease, distal hereditary motor neuropathy type VIIB, and frontotemporal dementia (FTD) [28,29,30]. Patient #6 showed a SPECT pattern consistent with AD, but her memory function was relatively preserved. Her dyspnea prompted us to suspect Perry syndrome or atypical Perry syndrome [29]. Recent studies also found *DCTN1* variants in FTD and Parkinson’s disease [31]. However, pathophysiological roles of *DCTN1* in these diseases have not yet been established [32]. The tentative diagnosis based upon the clinical finding was FTD, which might be partially supported by this genetic test.

Currently, there are no clear guidelines for genetic tests for dementia [33], yet there are several clinical genetic tests that are commercially available. According to the ACMG Guidelines for AD, the leading cause of dementia, symptomatic genetic tests can be performed in: (1) patients with autosomal dominant family history or early onset; or (2) sporadic or non-autosomal dominant family clustering. However, genetic tests only include *PS1*/*PS2*, *APP*, but not *APOE* [14]. Although more than 300 *PS1* variants have been found [34], the number of patients whose genetic tests were positive is small.

We were not able to detect any pathogenic variants in all patients. This could be partially because we used a gene panel with a limited number of genes, despite our gene panel covering most of the diseases associated with dementia. Although whole genomic/exonic sequencing (WGS/WES) could have detected novel gene variants, it is not straightforward in validating the pathogenicity of variants using experimental models [35]. We cannot test dementia in genetically engineered animal models since animals’ brain functions are profoundly different from human beings. In addition, it has been reported that discovery rates for feasible variants with WES and WGS might be similar to those with gene panels [10]. Although we have significantly advanced sequencing technologies, our knowledge to interpret VUS, in comparison, may still be behind [36,37,38].

There are several significant limitations in this study: (1) This is a single center study with a limited number of patients. However, positivity of genetic tests may not be associated with number of samples. (2) Although the age of onset was relatively high compared to previous studies, the average life expectancy in Japan is approximately 90 years. (3) We might have missed many unknown gene variants in association with neural functions from the gene panel since we were focusing on already known genes associated with neurodegenerative diseases. (4) Family members could not be genetically screened due to their wishes such as in family #6. In the other two families, the family members with dementia were already deceased.

## 5. Conclusions

We screened 18 Japanese dementia patients whose diagnoses were uncertain and who had positive family history of dementia using a gene panel of neurodegenerative diseases. We found three variants in three patients, although the significance of variants was uncertain. However, the variant in *DCTN1* might be association with the patient’s frontotemporal dementia. To link the results of the genetic test and our patients’ dementia phenotypes, further data accumulation and development of appropriate experimental models are warranted.

## Figures and Tables

**Table 1:** Clinical background. AD—Alzheimer’s Disease; MMSE—Mini-Mental State Examination; DM—diabetes mellitus; CAD—coronary artery disease; NP—not particular.

No.	Gender	Onset Age	Age	Past Medical History	Family Medical History	MMSE
1	M	74	76	DM, Hypertension	Father: Parkinson (77 y.o.), Mother: AD (67 y.o.)	20
2	F	73	75	Hyperlipidemia	Mother: AD	15
3	F	69	70	NP	Mother: AD (86 y.o.)	29
4	F	85	86	DM, After Traumatic brain hemorrhage	Older sister: Dementia	16
6	F	81	83	Cerebral aneurysm, Hypertension, CAD	Father and Sisters: Dementia	22
7	F	83	85	DM, Hypertension, Collagen disease	Mother: Dementia (60 y.o.)	23
9	F	68	71	NP	Mother: Dementia (60 y.o.); Older Brother: Dementia (60 y.o.)	25
11	F	54	61	NP	Grandfather: AD	21
12	F	69	74	NP	Mother: AD (70 y.o.)	19
13	F	61	63	NP	Father: Possible AD	18
14	M	72	73	After Brain tumor surgery	Mother: AD (90 y.o.)	24
15	F	78	80	NP	Father and Older Sister: Dementia	24
16	M	73	74	DM	Mother and Brothers: Dementia	24
17	F	87	87	NP	Mother: cerebral infarction; Older Sister: AD	28
18	F	81	90	NP	Older Sister: Dementia	21
19	M	81	81	DM, CAD	Mother: Dementia (80 y.o.)	30
21	F	74	74	NP	Mother: visual hallucination (70 y.o.); Father: cerebral stroke	19
23	F	63	65	Bipolar disorder, Pulmonary thrombosis	Mother: AD (69 y.o.); Grandmother AD	25

**Table 2:** Variants found in the three patients.

Patient.	Position	Transcript	Gene	Genotype	Coding	Protein	Sift	Polyphen	Grantham
1	chr7:143048939	NM_000083.2	*CLCN1*	G/A	c.2848G>A	p.Glu950Lys	0.01	0.826	56
2	chr1:237948187	NM_001035.2	*RYR2*	A/G	c.13175A>G	p.Lys4392Arg	-	0.001	26
6	chr2:74590743	NM_004082.4	*DCTN1*	C/T	c.3209G>A	p.Arg1070Gln	0.43	0.829	43

## Data Availability

The data presented in this study are available on request from the corresponding author. The data are not publicly available due to including confidential genetic data.

## References

[1] Tsugane S. (2020). Why has Japan become the world’s most long-lived country: Insights from a food and nutrition perspective. Eur. J. Clin. Nutr..

[2] Ganguli M., Hughes T.F., Jia Y., Lingler J., Jacobsen E., Chang C.H. (2020). Aging and Functional Health Literacy: A Population-based Study. Am. J. Geriatr. Psychiatry.

[3] Erkkinen M.G., Kim M.O., Geschwind M.D. (2018). Clinical Neurology and Epidemiology of the Major Neurodegenerative Diseases. Cold Spring Harb Perspect. Biol..

[4] Lam A.D., Deck G., Goldman A., Eskandar E.N., Noebels J., Cole A.J. (2017). Silent hippocampal seizures and spikes identified by foramen ovale electrodes in Alzheimer’s disease. Nat. Med..

[5] Jack C.R., Bennett D.A., Blennow K., Carrillo M.C., Dunn B., Haeberlein S.B., Holtzman D.M., Jagust W., Jessen F., Karlawish J. (2018). NIA-AA Research Framework: Toward a biological definition of Alzheimer’s disease. Alzheimers Dement..

[6] Humphries C., Kohli M.A., Whitehead P., Mash D.C., Pericak-Vance M.A., Gilbert J. (2015). Alzheimer disease (AD) specific transcription, DNA methylation and splicing in twenty AD associated loci. Mol. Cell. Neurosci..

[7] Goldman J.S., Hahn S.E., Catania J.W., LaRusse-Eckert S., Butson M.B., Rumbaugh M., Strecker M.N., Roberts J.S., Burke W., Mayeux R. (2011). Genetic counseling and testing for Alzheimer disease: Joint practice guidelines of the American College of Medical Genetics and the National Society of Genetic Counselors. Genet. Med..

[8] Kondo T., Imamura K., Funayama M., Tsukita K., Miyake M., Ohta A., Woltjen K., Nakagawa M., Asada T., Arai T. (2017). iPSC-Based Compound Screening and In Vitro Trials Identify a Synergistic Anti-amyloid beta Combination for Alzheimer’s Disease. Cell Rep..

[9] Goldman J.S., Van Deerlin V.M. (2018). Alzheimer’s Disease and Frontotemporal Dementia: The Current State of Genetics and Genetic Testing Since the Advent of Next-Generation Sequencing. Mol. Diagn. Ther..

[10] Koriath C.A.M., Kenny J., Ryan N.S., Rohrer J.D., Schott J.M., Houlden H., Fox N.C., Tabrizi S.J., Mead S. (2020). Genetic testing in dementia-utility and clinical strategies. Nat. Rev. Neurol..

[11] Valotassiou V., Malamitsi J., Papatriantafyllou J., Dardiotis E., Tsougos I., Psimadas D., Alexiou S., Hadjigeorgiou G., Georgoulias P. (2018). SPECT and PET imaging in Alzheimer’s disease. Ann. Nucl. Med..

[12] Barthel H., Schroeter M.L., Hoffmann K.T., Sabri O. (2015). PET/MR in dementia and other neurodegenerative diseases. Semin. Nucl. Med..

[13] Rothberg J.M., Hinz W., Rearick T.M., Schultz J., Mileski W., Davey M., Leamon J.H., Johnson K.L., Milgrew M.J., Edwards M. (2011). An integrated semiconductor device enabling non-optical genome sequencing. Nature.

[14] Reitz C. (2015). Genetic diagnosis and prognosis of Alzheimer’s disease: Challenges and opportunities. Expert Rev. Mol. Diagn..

[15] Richards S., Aziz N., Bale S., Bick D., Das S., Gastier-Foster J., Grody W.W., Hegde M., Lyon E., Spector E. (2015). Standards and guidelines for the interpretation of sequence variants: A joint consensus recommendation of the American College of Medical Genetics and Genomics and the Association for Molecular Pathology. Genet. Med..

[16] (2020). Genome Aggregation Database (gnomAD). https://gnomad.broadinstitute.org/.

[17] Landrum M.J., Kattman B.L. (2018). ClinVar at five years: Delivering on the promise. Hum. Mutat..

[18] (2020). National Center for Biotechnology Information: Online Mendelian Inheritance in Man1/18/2021. https://www.omim.org/.

[19] Adam M.P., Ardinger H.H., Pagon R.A., Wallace S.E., Bean L.J.H., Stephens K., Amemiya A. (2020). GeneReviews. https://www.ncbi.nlm.nih.gov/pubmed/20301295.

[20] A New Ontology Lookup Service at EMBL-EBI (2015). Proceedings of SWAT4LS International Conference 2015. https://www.ebi.ac.uk/ols/ontologies/mondo.

[21] (2020). Orphanet: The Portal for Rare Diseases and Orphan Drugs. https://www.orpha.net/consor/cgi-bin/index.php.

[22] (2020). National Center for Biotechnology Information: PubMed. https://www.ncbi.nlm.nih.gov/pubmed/.

[23] Kawamura M., Ohno S., Naiki N., Nagaoka I., Dochi K., Wang Q., Hasegawa K., Kimura H., Miyamoto A., Mizusawa Y. (2013). Genetic Background of Catecholaminergic Polymorphic Ventricular Tachycardia in Japan. Circ. J..

[24] Duno M., Colding-Jorgensen E., Adam M.P., Ardinger H.H., Pagon R.A., Wallace S.E., Bean L.J.H. (1993). Myotonia Congenita. GeneReviews(R).

[25] Liu J., Supnet C., Sun S., Zhang H., Good L., Popugaeva E., Bezprozvanny I. (2014). The role of ryanodine receptor type 3 in a mouse model of Alzheimer disease. Channels.

[26] Del Prete D., Checler F., Chami M. (2014). Ryanodine receptors: Physiological function and deregulation in Alzheimer disease. Mol. Neurodegener..

[27] Bussiere R., Lacampagne A., Reiken S., Liu X., Scheuerman V., Zalk R., Martin C., Checler F., Marks A.R., Chami M. (2017). Amyloid β production is regulated by β2-adrenergic signaling-mediated post-translational modifications of the ryanodine receptor. J. Biol. Chem..

[28] Caroppo P., Le Ber I., Clot F., Rivaud-Péchoux S., Camuzat A., De Septenville A., Boutoleau-Bretonnière C., Mourlon V., Sauvée M., Lebouvier T. (2014). DCTN1 mutation analysis in families with progressive supranuclear palsy-like phenotypes. JAMA Neurol..

[29] Konno T., Ross O.A., Teive H.A., Sławek J., Dickson D.W., Wszolek Z.K. (2017). DCTN1-related neurodegeneration: Perry syndrome and beyond. Park. Relat. Disord..

[30] Arakawa J., Hamabe A., Aiba T., Nagai T., Yoshida M., Toya T., Ishigami N., Hisadome H., Katsushika S., Tabata H. (2014). A novel cardiac ryanodine receptor gene (RyR2) mutation in an athlete with aborted sudden cardiac death: A case of adult-onset catecholaminergic polymorphic ventricular tachycardia. Hear. Vessel..

[31] Vilariño-Güell C., Wider C., Soto-Ortolaza A.I., Cobb S.A., Kachergus J.M., Keeling B.H., Dachsel J.C., Hulihan M.M., Dickson D.W., Wszolek Z.K. (2009). Characterization of DCTN1 genetic variability in neurodegeneration. Neurology.

[32] Procopio R., Gagliardi M., D’Amelio M., Brighina L., Nicoletti G., Morelli M., Bonapace G., Quattrone A., Annesi G. (2020). DCTN1 mutation analysis in Italian patients with PSP, MSA, and DLB. Neurobiol. Aging.

[33] Riarh A., Shelesky G., Hogan L. (2018). More Evidence Needed Regarding the Utility of Genetic Testing for Alzheimer Dementia. Am. Fam. Physician.

[34] Cacquevel M., Aeschbach L., Houacine J., Fraering P.C. (2012). Alzheimer’s Disease-Linked Mutations in Presenilin-1 Result in a Drastic Loss of Activity in Purified γ-Secretase Complexes. PLoS ONE.

[35] Drummond E., Wisniewski T. (2017). Alzheimer’s disease: Experimental models and reality. Acta Neuropathol..

[36] Fatkin D., Johnson R. (2020). Variants of Uncertain Significance and “Missing Pathogenicity”. J. Am. Heart Assoc..

[37] Hoffman-Andrews L. (2017). The known unknown: The challenges of genetic variants of uncertain significance in clinical practice. J. Law Biosci..

[38] Macklin S.K., Jackson J.L., Atwal P.S., Hines S.L. (2019). Physician interpretation of variants of uncertain significance. Fam. Cancer.

